# Triglyceride-glucose index and health outcomes: an umbrella review of systematic reviews with meta-analyses of observational studies

**DOI:** 10.1186/s12933-024-02241-y

**Published:** 2024-05-23

**Authors:** Jia-Li Yin, Jing Yang, Xin-Jian Song, Xue Qin, Yu-Jiao Chang, Xing Chen, Fang-Hua Liu, Yi-Zi Li, He-Li Xu, Yi-Fan Wei, Fan Cao, Xue-Li Bai, Lang Wu, Tao Tao, Jian Du, Ting-Ting Gong, Qi-Jun Wu

**Affiliations:** 1 Department of Clinical Epidemiology, Shengjing Hospital of China Medical University, Shenyang, China; 2 Clinical Research Center, Shengjing Hospital of China Medical University, Shenyang, China; 3 Department of Epidemiology, School of Public Health, China Medical University, Shenyang, China; 4 Department of Endocrinology, The Fourth Affiliated Hospital of China Medical University, Shenyang, China; 5 Department of Obstetrics and Gynecology, Shengjing Hospital of China Medical University, Shenyang, China; 6 Department of Obstetrics and Gynecology, The Fourth Affiliated Hospital of China Medical University, Shenyang, China; 7 Cancer Epidemiology Division, Population Sciences in the Pacific Program, University of Hawaii Cancer Center, University of Hawaii at Manoa, Honolulu, HI USA; 8 NHC Key Laboratory of Advanced Reproductive Medicine and Fertility, (China Medical University), National Health Commission, Shenyang, China

**Keywords:** Health, Meta-analysis, Observational study, Triglyceride-glucose index, Umbrella review

## Abstract

**Background:**

Numerous meta-analyses have explored the association between the triglyceride-glucose (TyG) index and diverse health outcomes, yet the comprehensive assessment of the scope, validity, and quality of this evidence remains incomplete. Our aim was to systematically review and synthesise existing meta-analyses of TyG index and health outcomes and to assess the quality of the evidence.

**Methods:**

A thorough search of PubMed, EMBASE, and Web of Science databases was conducted from their inception through to 8 April 2024. We assessed the quality of reviews using A Measurement Tool to Assess Systematic Reviews (AMSTAR) and the certainty of the evidence using the Grading of Recommendations, Assessment, Development and Evaluation (GRADE) system. This study was registered with PROSPERO (CRD: 42024518587).

**Results:**

Overall, a total of 95 associations from 29 meta-analyses were included, investigating associations between TyG index and 30 health outcomes. Of these, 83 (87.4%) associations were statistically significant (*P* < 0.05) according to the random effects model. Based on the AMSTAR tool, 16 (55.2%) meta-analyses were high quality and none was low quality. The certainty of the evidence, assessed by the GRADE framework, showed that 6 (6.3%) associations were supported by moderate-quality evidence. When compared with the lowest category of the TyG index, the risk of contrast-induced nephropathy (CIN) [relative risk (RR) = 2.25, 95%CI 1.82, 2.77], the risk of stroke in patients with diabetes mellitus (RR = 1.26, 95%CI 1.18, 1.33) or with acute coronary syndrome disease (RR = 1.56, 95%CI 1.06, 2.28), the prognosis of coronary artery disease (CAD)-non-fatal MI (RR = 2.02, 95%CI 1.32, 3.10), and the severity of CAD including coronary artery stenosis (RR = 3.49, 95%CI 1.71, 7.12) and multi-vessel CAD (RR = 2.33, 95%CI 1.59, 3.42) increased with high TyG index.

**Conclusion:**

We found that the TyG index was positively associated with many diseases including the risk of CIN and stroke, the prognosis of CAD, and the severity of CAD which were supported by moderate-quality evidence. TyG index might be useful to identify people at high-risk for developing these diseases.

**Supplementary Information:**

The online version contains supplementary material available at 10.1186/s12933-024-02241-y.

## Introduction

Insulin resistance (IR) is a pathological state marked by diminished cellular response to insulin, resulting in metabolic dysregulation that contributes to the onset of multiple chronic diseases [1]. Although the hyperinsulinemic-euglycemic clamp test is regarded as the gold standard for evaluating IR, its extensive clinical application is limited due to its high cost, time-consuming nature, and complex procedural requirements [2]. Besides, despite the homeostasis model assessment of IR index being the most readily available marker for assessing IR in clinical settings, it lacks convenience and economic viability for widespread clinical application [3]. However, triglyceride-glucose (TyG) index which obtained as the product of fasting triglycerides and plasma glucose levels serves as an arithmetic expression of IR [4]. This index is considered a more practical and reliable predictor of IR compared to the two aforementioned measurement tools and has been extensively utilized in clinical settings [3, 5]. Moreover, prior research indicates that the TyG index exhibits high sensitivity and specificity in diagnosing IR, offering benefits such as cost-effectiveness, simplicity of measurement, and potential for positive societal impact [6].

Recently, numerous studies have been performed to evaluate the associations between TyG index and a range of health outcomes like arterial stiffnes [7], heart failure [8], cardiovascular disease [9], and cancer [10]. Additionally, the TyG index may provide information for the risk assessment of diseases such as type 2 diabetes mellitus (T2DM) [11], atherosclerosis [12], and critical delirium [13]. With the rapid increase in primary studies on TyG index, the number of systematic reviews and meta-analyses are also accumulating [14–17]. However, these evidences have covered a wide range of health outcomes, and have inconsistent endpoints, making it challenging for researchers and policymakers. Consequently, it is imperative to objectively and succinctly synthesize these findings to facilitate evidence-based decision-making.

The methods of the umbrella review (UR) are standardized tool to provide a relatively comprehensive understanding of published systematic reviews with meta-analyses on a specific topic recently [18–20]. A unique feature of the included studies is that they are systematic reviews and meta-analyses with the aim of describing their quality, summarising and comparing their results, and discussing the strength of these results [20, 21]. Herein, we conducted an UR of systematic reviews with meta-analyses of observational studies to evaluate the associations between TyG index and health outcomes, which thus provide important information to decision makers for facilitating their comprehension.

## Methods

### Protocol registration

The UR was developed in accordance with the reporting guidance in the Preferred Reporting Items for Systematic Reviews and Meta-Analyses (PRISMA) reporting guideline and the Reporting guideline for overviews of reviews of healthcare interventions (PRIOR statement) (Additional file 1: Tables S1–S2) [22, 23]. We have registered the protocol of this UR in PROSPERO (https://www.crd.york.ac.uk/ PROSPERO, CRD: 42024518587).

### Literature search

We conducted a comprehensive literature search from inception to July 2023 by screening PubMed, EMBASE, and Web of Science of for systematic reviews with meta-analyses that investigated the association between TyG index and any human health outcome. Furthermore, one additional search was conducted on 8 April 2024 to ensure completeness. Our detailed search strategy was displayed in Additional file 1: Table S3. In addition, manual inspection of the bibliographies of the located systematic reviews and meta-analyses was conducted to uncover supplementary pertinent publications.

### Eligibility criteria

Two trained reviewers (JY and J-LY) screened independently the titles and abstracts retrieved from the database and conducted full-text screening to meet the inclusion criteria. Any discrepancy in the literature screening was resolved by a third reviewer (Q-JW). Studies were included according to the PECOS (Population, Exposure, Comparison, Outcome, Study design) strategy:

1. Population: adults (participants ≥ 18 years of age);
2. Exposure: TyG index;
3. Comparison: The lowest category of the TyG index;
4. Outcomes: any health outcome such as T2DM, cardiovascular disease, and cancer;
5. Study design: systematic review with meta-analyses of observational studies including cohort, case–control, and cross-sectional studies.

Studies were excluded on the basis of the following criteria:

1. Narrative reviews or systematic reviews that did not contain a quantitative synthesis;
2. Letters, comments or conference abstracts;
3. Systematic review with meta-analyses that included less than two primary studies;
4. Systematic reviews with meta-analyses not reporting comprehensive data for re-analysis, such as effect sizes [hazard ratio (HR), relative risk (RR), or odds ratio (OR)], 95% confidence intervals (CIs), the number of cases, and total population;
5. Systematic reviews with meta-analyses of animal studies and/or in vitro studies.

Furthermore, when more than one meta-analysis presented overlapping datasets on the same outcome, we chose the meta-analysis with the largest dataset [24]. If more than one comparison form was analyzed for a given outcome (e.g., dose–response analysis; highest vs. lowest, etc.), all comparison forms were included in our study [25].

### Data extraction

Two trained reviewers (JY and J-LY) collected the information from each eligible study independently. All disagreements were resolved by consultation with the senior reviewer (Q-JW). Extracted information from each eligible systematic review with meta-analysis were the first author, year of publication, journal, study design (such as cohort, case–control, and cross-sectional study), number of included studies, number of cases and participants, comparison, health outcomes type, meta-analysis metrics (HR, RR, or OR), and pooled effect. From each study included in the systematic review with meta-analysis, we also extracted the first author, publication year, number of cases and participants, comparison form (dose–response analysis; highest *vs.* lowest), specific risk estimates, and corresponding 95% CIs.

### Data analysis

For each association from eligible meta-analysis, we extracted the data from the original studies and recalculated the adjusted summary effect sizes and corresponding 95% CIs using random effects models [20, 26]. In each meta-analysis, we evaluated heterogeneity by using the I^2^ statistic, which ranges from 0 to 100% and represents the percentage of the total variation across studies that can be explained by heterogeneity. An I^2^ value exceeded 50% or 75% indicated significant or considerable heterogeneity, respectively [27].

In addition, to verify the robust of our results, a sensitivity analysis was conducted. If meta-analyses were excluded due to overlap, we did a re-analysis to verify whether their results were consistent with the main analysis [28]. All statistical analyses were conducted in STATA version 16.

### Quality assessment of evidence and methods

According to the Grading of Recommendations, Assessment, Development and Evaluation (GRADE), the evidence was graded as high, moderate, low, or very low quality to draw conclusions [29]. Observational studies initiating with low-quality evidence can be subjected to downgrades due to factors like risk of bias, inconsistency among results, indirect evidence, imprecision, and publication bias. We assigned risk of bias when the weight of studies less than 6 points assessed by Newcastle–Ottawa score exceed 50%. Imprecision was determined when the sample size was insufficient, and we judged imprecision when the events size less than 300. Indirectness reflects differences in study populations. We assigned inconsistency when heterogeneity measured by the I^2^ statistic was greater than 50% for binary outcomes, and a revised cut-off of I^2^ > 75% for high heterogeneity. The publication bias study was determined to have publication bias when the funnel plot was asymmetrical and the P values for Egger’s test was 0.10 [30]. Conversely, they may be upgraded in quality for reasons including a substantial effect size, the presence of a dose–response relationship, or the existence of plausible confounding that would, in all likelihood, underestimate the true effect [30]. We determined the dose–response relationship if the effect size increased proportionally with TyG index. The large magnitude was determined when the effect size above 2 or 5, and the plausible residual confounding supporting inferences regarding conclusions [30, 31].

Besides, the methodological quality of included studies was assessed by A Measurement Tool to Assess Systematic Reviews (AMSTAR) [32]. As a valid and dependable measurement tool in assessing the quality of systematic reviews and meta-analyses, AMSTAR assesses quality based on 11 aspects including a literature search, literature inclusion, data extraction, statistical analysis, and bias evaluation [33]. Two trained reviewers (JY and J-LY) completed the quality assessment of evidence and methods independently. Discrepancies were resolved through discussion with a senior reviewer (Q-JW).

## Results

### Literature review

**Fig. 1:**
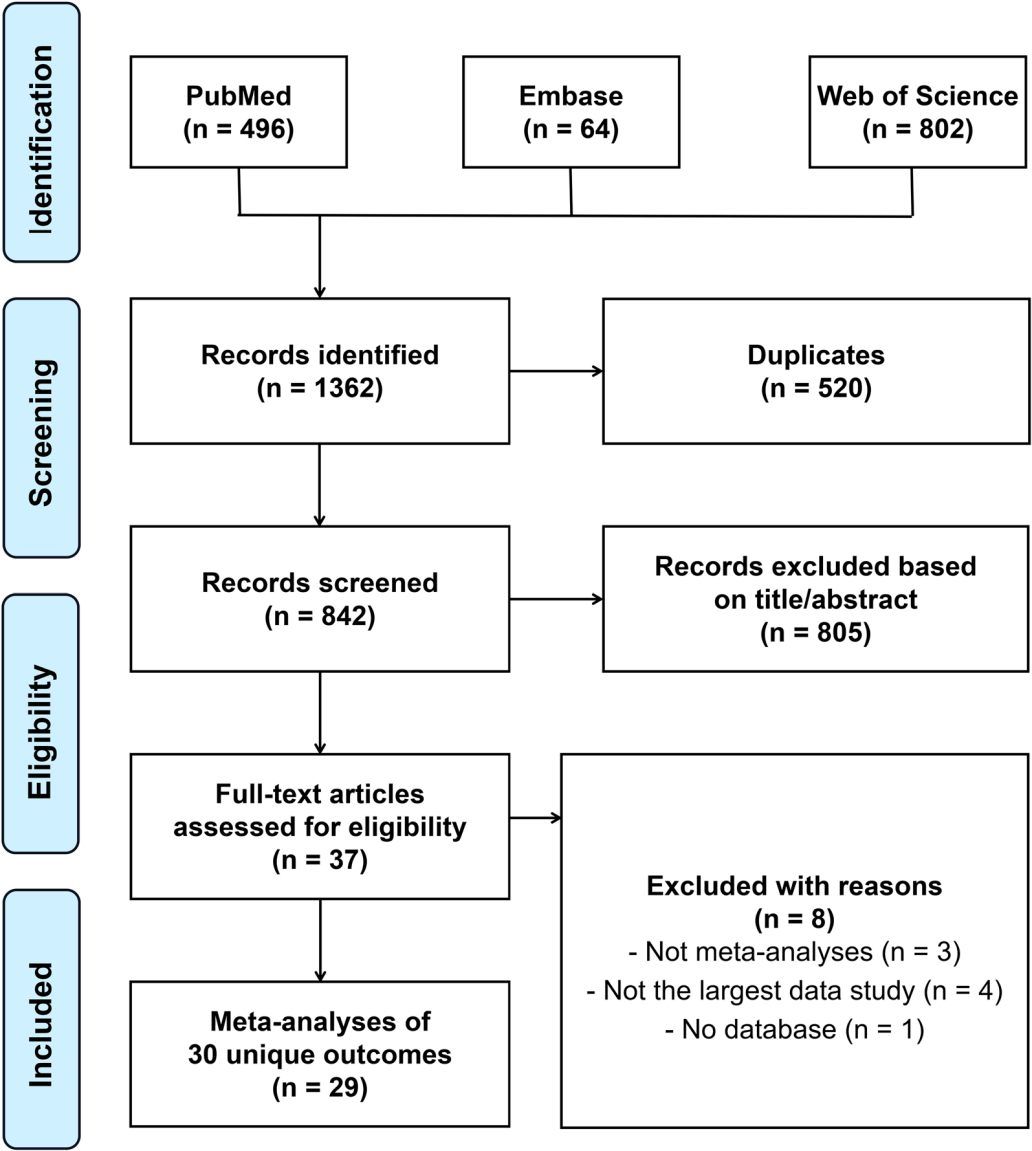
Flow diagram of the study selection process

Overall, the search retrieved 1362 records from PubMed, Web of Science, and Embase databases (Fig. 1). After removal of duplicates, 842 records were identified. After screening the titles and abstracts, 805 records were excluded. Eight records were further excluded based on full-text assessment (Additional file 1: Table S4). Ultimately, 29 [14–17, 34–58] articles were eligible to be included in the present UR (Fig. 1).

### Characteristics of the included meta-analyses

The eligible 29 articles described 95 associations published from 2020 to 2024, which estimated TyG index with four types of health outcomes, including disease risk (n = 59) (Table 1), prognosis (n = 31) (Table 2), severity (n = 3) (Table 3), and mortality (n = 2) (Table 4). We included 30 different health outcomes, such as cardiovascular disease, cancer, and so on (Additional file 1: Table S5). The majority of pooled analyses concerning the correlation between TyG index and coronary artery disease (CAD) (n = 26), followed by stroke (n = 7). We observed that the median number of primary studies was 4 (range 2–22), and the median number of participants was 11,615 (range 437–11,644,261) (Additional file 1: Table S5).

**Table 1:** Summary random-effects estimates with 95% confidence intervals of Triglyceride-glucose index and all health outcomes (Risk)

Outcomes (Risk)	Author, year, reference	Study characteristics	Comparison	Random effects (95% CI)	Random *P* value	I^2^	AMSTAR	GRADE
Arterial stiffness								
Arterial stiffness	Sajdeya et al. 2022 [34]	Female	Highest vs. lowest	1.84 (1.32, 2.55)	2.825E−4	0	5	Low
Arterial stiffness	Sajdeya et al. 2022 [34]	Male	Highest vs. lowest	1.88 (1.16, 3.06)	0.011	82.1	5	Very low
Arterial stiffness	Liu et al. 2023 [35]	–	Per 1-unit increment	1.51 (1.35, 1.69)	1.888E−12	81.7	10	Very low
Arterial stiffness	Liu et al. 2023 [35]	–	Highest vs. lowest	1.83 (1.55, 2.17)	2.844E−12	68.4	10	Very low
Atherosclerotic cardiovascular diseases								
Atherosclerotic cardiovascular diseases	Ding et al. 2021 [36]	–	Highest vs. lowest	1.61 (1.29, 2.01)	2.317E−05	79.9	7	Very low
Atherosclerotic cardiovascular diseases	Ding et al. 2021 [36]	–	Per 1-unit increment	1.28 (1.13, 1.45)	1.380E−04	61.7	7	Very low
Atherosclerotic cardiovascular diseases	Ding et al. 2021 [36]	Male	Highest vs. lowest	1.44 (1.14, 1.83)	3.000E−03	77.5	7	Very low
Atherosclerotic cardiovascular diseases	Ding et al. 2021 [36]	Female	Highest vs. lowest	1.65 (1.13, 2.42)	9.000E−03	82.4	7	Very low
Atherosclerotic cardiovascular diseases	Ding et al. 2021 [36]	Diabetes mellitus	Highest vs. lowest	1.66 (0.94. 2.91)	7.900E−02	63.8	7	Very low
Atherosclerotic cardiovascular diseases	Ding et al. 2021 [36]	Non-diabetes mellitus	Highest vs. lowest	1.52 (1.11, 2.08)	9.000E−03	86.9	7	Very low
Atrial fibrillation								
Atrial fibrillation	Azarboo et al. 2024 [52]	Patients with hypertrophic obstructive cardiomyopathy after septal myectomy	Experimental vs. Control	0.99 (0.77, 1.21)	3.731E−18	10.8	8	Very low
Atrial fibrillation	Azarboo et al. 2024 [52]	Patients with atrial fibrillation who underwent radiofrequency catheter ablation	Experimental vs. Control	1.23 (0.98, 1.49)	1.291E−21	48.4	8	Very low
Atrial fibrillation	Azarboo et al. 2024 [52]	Patients diagnosed with Non-alcoholic fatty liver disease by ultrasound	Experimental vs. Control	1.45 (0.41, 3.31)	0.127	99.6	8	Very low
Cancer								
Cancer	Wang et al. 2023 [17]	–	Highest vs. lowest	1.70 (1.14, 2.54)	9.000E−03	93.2	7	Very low
Cardiovascular disease								
Cardiovascular disease	Liu et al. 2022 [37]	–	Highest vs. lowest	1.47 (1.23, 1.74)	1.393E−05	82.0	10	Very low
Cardiovascular disease	Liu et al. 2022 [37]	–	Per 1-unit increment	1.23 (1.12, 1.35)	1.582E−05	88.9	10	Very low
Cerebrovascular disease								
Cerebrovascular disease	Yan et al. 2022 [38]	–	Case vs. control	1.14 (1.05, 1.23)	1.000E−03	70.2	8	Very low
Cerebrovascular disease	Yan et al. 2022 [38]	–	High vs. low index	1.26 (1.18, 1.35)	1.160E−10	80.4	8	very low
Chronic kidney disease								
Chronic kidney disease	Ren et al. 2023 [39]	–	Highest vs. lowest	1.47 (1.32, 1.63)	1.499E−12	50.4	7	Very low
Cognitive impairment								
Cognitive impairment	Wang et al. 2023 [55]	General population	Highest vs. lowest	2.32 (1.39, 3.87)	1.000E−03	84.7	7	Very low
Cognitive impairment	Wang et al. 2023 [55]	Patients with Cerebral small vessel disease	Highest vs. lowest	3.38 (1.67, 6.84)	1.000E−03	81.6	7	Very low
Coronary artery calcification								
Coronary artery calcification	Liu et al. 2023 [35]	–	Highest vs. lowest	1.66 (1.51, 1.83)	7.630E−25	0	10	Low
Coronary artery calcification	Liu et al. 2023 [35]	–	Per 1-unit increment	1.73 (1.36, 2.20)	7.593E−06	50.8	10	Very low
Coronary artery disease								
Coronary artery disease	Ding et al. 2021 [36]	–	Highest vs. lowest	1.95 (1.47, 2.59)	3.699E−06	91.8	7	Very low
Coronary artery disease	Liu et al. 2022 [37]	–	Per 1-unit increment	1.35 (1.02, 1.79)	3.600E−02	93.8	10	Very low
Coronary artery disease	Liang et al. 2023 [40]	Without coronary atherothrombotic disease or with suspected	Highest vs. lowest	1.94 (1.20, 3.14)	7.000E−03	91.3	8	Very low
Contrast-inducednephropathy								
Contrast-induced nephropathy	Chang et al. 2023 [53]	Non-diabetes mellitus patients with non-ST-segment elevation myocardial infarction	High TyG index vs. low TyG index	2.25 (1.82, 2.77)	3.541E−14	35.4	7	Moderate
Dementia								
Dementia	Wang et al. 2023 [55]	General population	Highest vs. lowest	1.14 (1.12, 1.16)	9.32E−49	0	7	Low
Dementia	Wang et al. 2023 [55]	General population	Highest vs. lowest	1.37 (1.03, 1.83)	0.031	0	7	Low
Diabetic retinopathy								
Diabetic retinopathy	Zhou et al. 2023 [54]	Type 2 diabetes mellitus patients	Highest vs. lowest	2.35 (1.31, 4.18)	4.000E−03	89.7	8	Very low
Diabetic retinopathy	Zhou et al. 2023 [54]	Type 2 diabetes mellitus patients	Highest vs. lowest	1.48 (1.12, 1.97)	6.000E−03	83.5	8	Very low
Gestational diabetes mellitus								
Gestational diabetes mellitus	Song et al. 2022 [41]	Pregnancy women	Highest vs. lowest	2.52 (1.33, 4.76)	4.000E−03	64.5	7	Very low
Heart failure								
Heart failure	Khalaji et al. 2023 [51]	Population-based cohort aged 45 to 64 years	Highest vs. lowest	1.21 (1.13, 1.30)	2.598E−07	21.4	7	Low
Heart failure	Khalaji et al. 2023 [51]	Population-based adult cohort (Kailuan cohort)	1-unit increase	1.16 (1.11, 1.22)	9.438E−11	0.6	7	Low
Hypertension								
Hypertension	Xu et al. 2023 [50]	–	Highest vs. lowest	1.36 (1.28, 1.45)	1.143E−21	67.7	9	Very low
Hypertension	Xu et al. 2023 [50]	Male	Highest vs. lowest	1.36 (1.1, 1.53)	3.207E−07	77.8	9	Very low
Hypertension	Xu et al. 2023 [50]	Female	Highest vs. lowest	1.46 (1.26, 1.70)	5.866E−07	83.1	9	Very low
Ischemic stroke								
Ischemic stroke	Yang et al. 2023 [42]	–	Highest vs. lowest	1.37 (1.22, 1.54)	1.076E−07	74.2	8	Very low
Metabolic dysfunction-associated fatty liver disease								
Metabolicdysfunction-associated fatty liver disease	Wang et al. 2022 [57]	–	Case vs. control	5.56 (4.41,7.02)	1.660E−47	98.6	7	Very low
Metabolic syndrome								
Metabolic syndrome	Nabipoorashrafi et al. 2022 [58]	–	Case vs. control (with and without metabolic syndrome)	0.84 (0.70, 1.00)	5.100E−02	99.2	8	Very low
Metabolic syndrome	Nabipoorashrafi et al. 2022 [58]	Female	Case vs. control	0.82 (0.79, 0.86)	1.471E−22	0	8	Very low
Metabolic syndrome	Nabipoorashrafi et al. 2022 [58]	Male	Case vs. control	0.80 (0.69, 0.92)	3.000E−03	89.9	8	Very low
Myocardial infarction								
Myocardial infarction	Liu et al. 2022 [37]	–	Highest vs. lowest	1.36 (1.18, 1.56)	2.736E−05	35.0	10	Low
Nonalcoholic fatty liver disease								
Nonalcoholic fatty liver disease	Ling et al. 2023 [43]	–	Per 1-unit increment	2.84 (2.02, 4.01)	2.554E−09	98.2	10	Very low
Nonalcoholic fatty liver disease	Beran et al. 2022 [44]	–	Highest vs. lowest	4.20 (2.69, 6.57)	3.120E−10	0	7	Very low
Nonalcoholic fatty liver disease	Beran et al. 2022 [44]	Diabetes mellitus	Highest vs. lowest	7.52 (3.82, 14.82)	5.471E−09	89.9	7	Very low
Nonalcoholic fatty liver disease	Beran et al. 2022 [44]	Non-Diabetes mellitus	Highest vs. lowest	4.68 (3.02, 7.24)	4.448E−12	92.4	7	Very low
Obstructive sleep apnea								
Obstructive sleep apnea	Behnoush et al. 2024 [56]	Non-obese, non diabetic patients with symptoms suggestive for obstructive sleep apnea	Obstructive sleep apnea group vs. control group	0.86 (0.57, 1.15)	5.94E−09	34.9	8	Low
Stroke								
Stroke	Liao et al. 2023 [15]	–	Per 1-unit increment	1.13 (1.08, 1.18)	1.776E−08	0	7	Low
Stroke	Feng et al. 2022 [45]	–	Highest vs. lowest	1.28 (1.23, 1.33)	2.530E−33	5.2	8	Low
Stroke	Feng et al. 2022 [45]	Diabetes mellitus	Highest vs. lowest	1.26 (1.18, 1.33)	3.603E−14	0	8	Moderate
Stroke	Feng et al. 2022 [45]	Non-diabetes mellitus	Highest vs. lowest	1.21 (1.04, 1.40)	1.200E−02	78.3	8	Very low
Stroke	Feng et al. 2022 [45]	Acute coronary syndrome patients	Highest vs. lowest	1.56 (1.06, 2.28)	2.300E−02	0	8	Moderate
Stroke—cerebral infarction	Feng et al. 2022 [45]	–	Highest vs. lowest	1.51 (1.29, 1.77)	2.924E−07	14.4	8	Low
Stroke—non-fatal stroke	Feng et al. 2022 [45]	–	Highest vs. lowest	1.41 (1.06, 1.86)	1.700E−02	0	8	Low
Type 2 diabetes mellitus		–						
Type 2 diabetes mellitus	Silva et al. 2020 [46]	Female	Highest vs. lowest	2.11 (1.61, 2.76)	6.035E−08	88.9	8	Very low
Type 2 diabetes mellitus	Silva et al. 2020 [46]	Male	Highest vs. lowest	1.59 (1.35, 1.88)	3.124E−08	53.9	8	Very low
Type 2 diabetes mellitus	Silva et al. 2020 [46]	–	Exposed vs. unexposed	3.12 (2.31, 4.21)	1.288E−13	86.5	8	Very low
Type 2 diabetes mellitus	Pranata et al. 2021 [14]	–	Highest vs. lowest	3.53 (2.75, 4.54)	6.929E−23	83.7	8	Very low

AMSTAR, A Measurement Tool to Assess Systematic Reviews; CI: confidence interval; GRADE, Grading of Recommendations Assessment, Development, and Evaluation

**Table 2:** Summary random-effects estimates with 95% confidence intervals of Triglyceride-glucose index and all health outcomes (Prognosis)

Outcomes (Prognosis)	Author, year, reference	Study characteristics	Comparison	Random effects (95% CI)	Random *P* value	I^2^	AMSTAR	GRADE
Coronary artery calcification								
Coronary artery calcification	Liu et al. 2023 [35]	–	Highest vs. lowest	1.66 (1.21, 2.27)	1.778E−03	0	10	Low
Coronary artery calcification	Liu et al. 2023 [35]	–	Per 1-unit increment	1.47 (1.29, 1.68)	1.626E−08	41	10	Low
Coronary artery disease								
Coronary artery disease prognosis—all cause death	Luo et al. 2021 [16]	Coronary Atherothrombotic Disease patients	Highest vs. lowest	1.33 (0.82, 2.16)	2.460E−01	65.5	8	Very low
Coronary artery disease prognosis—all cause death	Sun et al. 2024 [49]	Following percutaneous coronary intervention	Highest vs. lowest	1.31 (0.53–3.22)	5.560E−01	50.9	9	Low
Coronary artery disease prognosis—all cause death	Sun et al. 2024 [49]	Following percutaneous coronary intervention	Per 1 standard unit	0.94 (0.29–3.13)	9.240E−01	74	9	Very low
Coronary artery disease prognosis—cardiovascular death	Luo et al. 2021 [16]	Coronary atherothrombotic disease patients	Highest vs. lowest	1.87 (0.90, 3.88)	9.400E−02	68.2	8	Very low
Coronary artery disease prognosis—major adverse cardiovascular events	Liang et al. 2023 [40]	Acute coronary syndrome patients	Highest vs. lowest	2.09 (1.68, 2.62)	7.459E−11	87.4	8	Very low
Coronary artery disease prognosis—major adverse cardiovascular events	Li et al. 2021 [47]	Acute coronary syndrome patients with Diabetes mellitus	Highest vs. lowest	1.98 (1.31, 2.99)	1.000E−03	86.7	7	Very low
Coronary artery disease								
Coronary artery disease prognosis—major adverse cardiovascular events	Li et al. 2021 [47]	Acute coronary syndrome patients without diabetes mellitus	Highest vs. lowest	1.62 (1.02, 2.57)	4.200E−02	86.4	7	Very low
Coronary artery disease prognosis—major adverse cardiovascular events	Luo et al. 2021 [16]	Coronary Atherothrombotic disease patients	Per 1-unit increment	1.70 (1.37, 2.10)	1.389E−06	85.5	8	Very low
Coronary artery disease prognosis—major adverse cardiovascular events	Liang et al. 2023 [40]	Acute coronary syndrome patients	Per 1-unit increment	2.28 (1.44, 3.62)	4.638E−04	94.7	8	Very low
Coronary artery disease prognosis—major adverse cardiovascular events	Liang et al. 2023 [40]	Chronic coronary syndrome and stable coronary atherothrombotic disease patients	Highest vs. lowest	1.24 (0.97, 1.59)	9.100E−02	84.3	8	Very low
Coronary artery disease prognosis—major adverse cardiovascular events	Liang et al. 2023 [40]	Chronic coronary syndrome and stable coronary atherothrombotic disease patients	Per 1-unit increment	1.49 (1.21, 1.84)	1.504E−04	74.5	8	Very low
Coronary artery disease								
Coronary artery disease prognosis—major adverse cardiovascular events	Luo et al. 2021 [16]	Coronary atherothrombotic disease patients	Highest vs. lowest	2.14 (1.69, 2.71)	2.823E−10	82.6	8	Very low
Coronary artery disease prognosis—major adverse cardiovascular events	Sun et al. 2024 [49]	Following percutaneous coronary intervention	Highest vs. lowest	2.04 (1.65–2.52)	5.754E−11	77.1	9	Very low
Coronary artery disease prognosis—major adverse cardiovascular events	Sun et al. 2024 [49]	Following percutaneous coronary intervention	Per 1 standard unit	1.82 (1.34–2.46)	1.096E−04	91.7	9	Very low
Coronary artery disease prognosis—major adverse cardiovascular events	Sun et al. 2024 [49]	In the post-percutaneous coronary intervention population with diabetes	Highest vs. lowest	2.28 (1.58–3.28)	9.272E−06	78.2	9	Very low
Coronary artery disease prognosis—major adverse cardiovascular events	Sun et al. 2024 [49]	In the post-percutaneous coronary intervention population without diabetes	Highest vs. lowest	2.43 (1.74–3.38)	1.640E−07	55.9	9	Very low
Coronary artery disease prognosis—myocardial infarction	Luo et al. 2021 [16]	Coronary atherothrombotic disease patients	Highest vs. lowest	1.90 (1.46, 2.46)	1.734E−06	0	8	Low
Coronary artery disease								
Coronary artery disease prognosis—non-fatal myocardial infarction	Sun et al. 2024 [49]	Following percutaneous coronary intervention	Highest vs. lowest	2.02 (1.32–3.10)	1.000E−03	0	9	Moderate
Coronary artery disease prognosis—non-fatal myocardial infarction	Sun et al. 2024 [49]	Following percutaneous coronary intervention	Per 1 standard unit	2.56 (1.49, 4.41)	1.000E−03	63.4	9	Very low
Coronary artery disease prognosis—revascularization	Luo et al. 2021 [16]	Coronary atherothrombotic disease patients	Highest vs. lowest	2.60 (1.76, 3.84)	1.663E−06	71.1	8	Very low
Coronary artery disease prognosis—revascularization	Sun et al. 2024 [49]	Following percutaneous coronary intervention	Highest vs. lowest	2.61 (1.47, 4.65)	1.000E−03	83.6	9	Very low
Coronary artery disease prognosis—revascularization	Sun et al. 2024 [49]	Following percutaneous coronary intervention	Per 1 standard unit	2.06 (1.21, 3.50)	8.000E−03	89.8	9	Very low
Coronary artery disease prognosis—stroke	Luo et al. 2021 [16]	Coronary atherothrombotic disease patients	Highest vs. lowest	1.56 (1.06, 2.28)	2.300E−02	0	8	Low
Ischemic stroke								
Ischemic stroke prognosis—all-cause mortality	Ma et al. 2022 [48]	Acute Ischemic stroke patients	Highest vs. lowest	1.60 (1.19, 2.15)	2.000E−03	78.2	7	Very low
Ischemic stroke prognosis—mortality	Yang et al. 2023 [42]	Ischemic stroke patients	Highest vs. lowest	1.40 (1.14, 1.71)	1.000E−03	70.7	8	Very low
Ischemic stroke prognosis—neurological worsening	Yang et al. 2023 [42]	Ischemic stroke patients	Highest vs. lowest	1.76 (0.79, 3.95)	1.687E−01	76.6	8	Very low
Ischemic stroke prognosis—poor functional outcome	Ma et al. 2022 [48]	Acute Ischemic stroke patients	Highest vs. lowest	1.37 (1.11, 1.69)	4.000E−03	71.3	7	Very low
Ischemic stroke prognosis—poor functional outcome	Yang et al. 2023 [42]	Ischemic stroke patients	Highest vs. lowest	1.12 (0.88, 1.43)	3.580E−01	77.3	8	Very low
Ischemic stroke prognosis—stroke recurrence	Yang et al. 2023 [42]	Ischemic stroke patients	Highest vs. lowest	1.50 (1.19, 1.89)	1.000E−03	56.2	8	Very low

AMSTAR, A Measurement Tool to Assess Systematic Reviews; CI: confidence interval; GRADE, Grading of Recommendations Assessment, Development, and Evaluation

**Table 3:** Summary random-effects estimates with 95% confidence intervals of Triglyceride-glucose index and all health outcomes (Severity)

Outcomes (Severity)	Author, year, reference	Study characteristics	Comparison	Random effects (95% CI)	Random *P* value	I^2^	AMSTAR	GRADE
Coronary artery disease								
Coronary artery disease severity—coronary artery palque progress	Liang et al. 2023 [40]	Coronary atherothrombotic disease patients	High vs. low index	1.68 (1.28, 2.19)	1.639E−04	0	8	Low
Coronary artery disease severity—coronary artery stenosis	Liang et al. 2023 [40]	Coronary atherothrombotic disease patients	High vs. low index	3.49 (1.71, 7.12)	5.975E−04	0	8	Moderate
Coronary artery disease severity—multi-vessle coronary artery disease	Liang et al. 2023 [40]	Coronary atherothrombotic disease patients	High vs. low index	2.33 (1.59, 3.42)	1.576E−05	0	8	Moderate

AMSTAR, A Measurement Tool to Assess Systematic Reviews; CI: confidence interval; GRADE, Grading of Recommendations Assessment, Development, and Evaluation

**Table 4:** Summary random-effects estimates with 95% confidence intervals of Triglyceride-glucose index and all health outcomes (Mortality)

Outcomes (Mortality)	Author, year, reference	Study characteristics	Comparison	Random effects (95% CI)	Random *P* value	I^2^	AMSTAR	GRADE
All-cause mortality								
All-cause mortality	Liu et al. 2022 [37]	–	Highest vs. lowest	1.08 (0.92, 1.28)	3.500E−01	86.9	10	Very low
Cardiovascular death								
Cardiovascular death	Liu et al. 2022 [37]	–	Highest vs. lowest	1.10 (0.82, 1.47)	5.310E−01	75.6	10	Very low

AMSTAR, A Measurement Tool to Assess Systematic Reviews; CI: confidence interval; GRADE, Grading of Recommendations Assessment, Development, and Evaluation

### Methodological quality of included meta-analyses

**Fig. 2:**
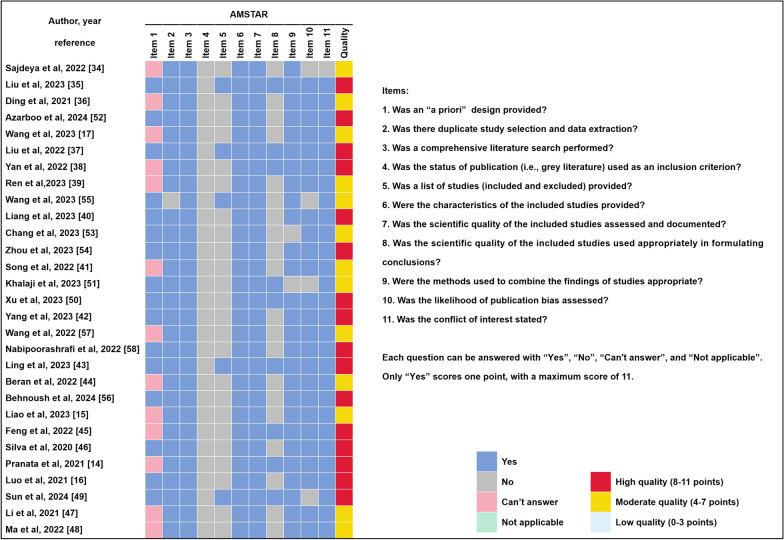
Methodological quality assessment of the included articles with AMSTAR

The AMSTAR scores of these articles ranged from 5 to 10, with a median score of 8. Among them, 16 (55.2%) and 13 articles (44.8%) were designated as high and moderate quality, respectively (Fig. 2). AMSTAR, assessment of multiple systematic reviews.

### Summary findings and heterogeneity of the included meta-analyses

Among the 95 associations, the magnitude of the observed summary random-effects estimates ranged from 0.80 to 7.52 (Tables 1, 2, 3, 4). A total of 83 associations (87.4%) showed statistical significance at the *P* < 0.05 level based on random effects model (Additional file 1: Table S5). Out of the 95 associations, 33 (34.7%) displayed low heterogeneity (*I*^2^ < 50%), 18 (19.0%) exhibited high heterogeneity (*I*^2^ = 50–75%), and 44 (46.3%) presented very high heterogeneity (*I*^2^ > 75%) (Tables 1, 2, 3, 4).

### Certainty of evidence

The certainty of the evidence assessed using the GRADE framework revealed that 6 (6.3%) associations were supported by moderate-quality evidence. These associations included the risk of contrast-induced nephropathy (CIN) in non-diabetes mellitus patients with non-ST-segment elevation myocardial infarction (MI) after percutaneous coronary intervention (PCI) (high *vs.* low, RR = 2.25, 95%CI 1.82, 2.77), the risk of stroke in patients with diabetes mellitus (highest *vs.* lowest, RR = 1.26, 95%CI 1.18, 1.33), and the risk of stroke in patients with acute coronary syndrome (ACS) disease (highest *vs.* lowest, RR = 1.56, 95%CI 1.06, 2.28), the prognosis of CAD—non-fatal MI after PCI (highest *vs.* lowest, RR = 2.02, 95%CI 1.32, 3.10), the severity of CAD—Coronary artery stenosis (high *vs.* low, RR = 3.49, 95%CI 1.71, 7.12), the severity of CAD—Multi-vessel CAD (high *vs.* low, RR = 2.33, 95%CI 1.59, 3.42) (Tables 1, 2, 3, 4 and Additional file 1: Table S5). The remaining associations were categorized as low-quality evidence (n = 18, 19.0%) and very low-quality evidence (n = 71, 74.7%). The most common reason for downgrading was inconsistency (68 associations), followed by publication bias (25 associations) and risk of bias (4 associations) (Additional file 1: Table S6).

### Sensitivity analyses

Sensitivity analysis was conducted for meta-analyses excluded due to overlap, with 15 associations qualifying for this analysis (Additional file 1: Table S7). The outcomes evaluated in these meta-analyses included eight outcomes [arterial stiffness, CAD, hypertension, gestational diabetes mellitus, major adverse cardiovascular events (MACEs), nonalcoholic fatty liver disease, stroke, and T2DM]. The two meta-analyses that elevated the level of evidence from very low to low pertained to the association between the TyG index and the risk of hypertension in females, as well as the association between the TyG index and the risk of MACE in patients with ACS. Besides, the evidence of a meta-analysis increased from low to moderate with regard to the association between TyG index and risk of stroke. However, the evidence provided by one meta-analyse decreased to very low from low, and 11 meta-analyses remained unchanged (Additional file 1: Table S7).

## Discussion

### Main findings

This UR first summarized and evaluated the evidence of TyG index with diverse health outcomes. According to the criteria of GRADE, six associations consisting of the risk of CIN in non-diabetic patients with non-ST-elevation MI after PCI (high *vs.* low), the risk of stroke in patients with diabetes mellitus (highest *vs.* lowest), the risk of stroke in patients with ACS disease (highest *vs.* lowest), the prognosis of CAD—non-fatal MI after PCI (highest *vs.* lowest), the the severity of CAD—Coronary artery stenosis (high *vs.* low), the severity of CAD—Multi-vessel CAD (high *vs.* low) were graded as moderate-quality level.

We found that a high TyG index was associated with an increased risk of CIN in non-diabetic patients with non-ST-segment elevation MI after PCI with moderate-quality evidence. However, the diagnostic accuracy of the TyG index for CIN after PCI is moderate and the TyG index should not be used alone for CIN screening [53]. Contrast-induced acute kidney injury (CI-AKI), also known as CIN, is an acute kidney injury caused by the use of iodinated contrast media and has emerged as one of the major complications associated with coronary angiography and interventional cardiology [59]. A high TyG index is significantly and independently associated with the incidence of CIN in patients with non-ST-elevation ACS undergoing initial drug-eluting stent (DES) implantation [60]. Routine preoperative assessment of the TyG index may alleviate CIN and the TyG index is a potential target for intervention to prevent CIN [60]. In addition, a study of patients with suspected CAD undergoing coronary angiography or PCI found that the incidence of CI-AKI increased sharply with increasing TyG [61]. Univariate and multivariate analysis identified TyG as an independent risk factor for CI-AKI [61]. Furthermore, a higher TyG index was found to increase the incidence of CIN in non-diabetic, non-ST-elevation acute MI patients undergoing coronary angiography and is an independent risk factor for the development of CIN [62]. The results of these studies have been consistent with our findings.

Stroke was the outcome for two other associations that were supported by moderate-quality evidence. Subgroup analyses showed that the baseline TyG index was positively associated with the risk of stroke, regardless of whether comorbidity with diabetes or not. There was a linear trend between the TyG index and the risk of stroke in diabetic patients, but there was insufficient data to perform a dose–response analysis in non-diabetic patients [45]. Findings from a cohort study of 5014 seemingly healthy individuals found that high TyG index was associated with an increased risk of cardiovascular disease, including stroke, irrespective of diabetic status [63]. Nested case–control study of 1282 patients with T2DM and stable CAD found positive association between TyG index and future cardiovascular events, including stroke [64]. Liu et al. [65] demonstrated that the TyG index could predict the risk of stroke in non-diabetic populations. Additionally, alterations in the TyG index exhibited a more robust correlation with stroke events in non-diabetic individuals, possibly attributable to the application of glucose-lowering medications mitigating stroke risk [66]. Meanwhile, elevated TyG index related to the increased risk of stroke in ACS patients has been confirmed by many studies [67–69]. Of note, the TyG index was positively associated with an increased risk of MACE including stroke in a cohort study of 2531 consecutive diabetic patients [67]. The researchers concluded that the TyG index serves as an independent predictor for the manifestation of MACE in individuals with diabetes and ACS [67]. Besides, Ma et al. demonstrated that, following adjustment for confounding variables, the TyG index was significantly associated with a heightened risk of cardiovascular events in patients with T2DM and ACS who underwent PCI [68]. A retrospective study of 1158 patients with ACS who had previously undergone coronary artery bypass grafting and were undergoing PCI found that those with a higher TyG index had a significantly higher incidence of stroke than those with a lower TyG index [69].

Our research showed that those with the highest TyG index had a significantly higher risk of non-fatal MI after PCI than those with the lowest TyG index, supported by moderate-quality evidence. This result is in line with several previous surveys [70–72]. The study by Sun et al. [70] investigating the impact of the TyG index on the prognosis of patients with ischemic heart failure undergoing PCI showed that the incidence of non-fatal MI increased significantly with increasing TyG quartiles. The independent association between TyG index and increased risk of non-fatal MI was confirmed [70]. Besides, a retrospective study showed that in patients with T2DM and non-ST-segment elevation ACS treated with PCI, the incidence of non-fatal MI was significantly increased in patients with a higher TyG index [71]. In addition, a study of ACS patients without diabetes mellitus who underwent emergency PCI with DES found that the TyG index may be an independent predictor of major adverse cardiovascular and cerebrovascular event (MACCE). This study had a median follow-up of 47 months and selected MACCE as the observed endpoint [72]. However, there was no significant difference in the incidence of non-fatal MI in patients with high TyG compared with the low group [72]. This may be due to the insufficient sample size of this study (only 10 for non-fatal MI).

Our UR provided moderate-quality evidence supporting the positive association between TyG index and the severity of coronary artery stenosis in CAD patients, aligning with the findings of several preceding studies [73–75]. For example, a retrospective study conducted by Xu et al. [73] showed a significant positive correlation between the TyG index and severe coronary artery stenosis in patients with hypertension and CAD. Additionally, the TyG index has been identified as an independent risk factor for the severity of coronary artery stenosis [73]. Besides, a cross-sectional study demonstrated that the TyG index might serve as a marker for IR [74]. An elevated TyG index may be indicative of patients at heightened risk for coronary artery stenosis and is linked to the extent of arterial stenoses [74]. In addition, a retrospective analysis involving 2,952 patients revealed that the TyG index serves as a predictor for the severity of coronary artery stenosis in individuals with premature cardiovascular artery disease (PCAD), thus establishing its utility as both a diagnostic and risk marker for coronary artery stenosis in PCAD patients [75].

Our results showed that the TyG index in CAD patients is positively correlated with CAD severity-Multi-vessle CAD severity, which is supported by moderate-quality evidence. This finding was consisted with results of several previous studies [76–78]. For instance, a retrospective analysis revealed that patients with multivessel CAD exhibited a significantly increased TyG index compared to individuals with single-vessel CAD, suggesting that the TyG index is associated with the severity of CAD and also constitutes an independent risk factor for multivessel CAD [76]. Furthermore, an increased TyG index was associated with an increased risk of multivessel CAD in a study of patients with CAD [77]. What is more, a multicentre retrospective study of patients with CAD showed that an elevated TyG index was associated with an increased risk of multiple coronary atherosclerosis [78].

Our study confirms the association between TyG and a range of health outcomes. The cost-effective measurement of TyG indices has important clinical implications for the early identification of individuals at risk for these diseases and for improving risk stratification and treatment management [79]. Based on the dynamic nature of disease progression and the TyG index, it is suggested that clinical assessment of the TyG index at a single time point has limitations [80]. Primary care physicians additionally need to be aware of this when making judgements using the TyG index.

### Strengths and limitations

This study constitutes the first systematic appraisal of the association between the TyG index and diverse health outcomes, integrating findings from meta-analyses of extant observational studies and utilizing the well-recognized GRADE criteria for the assessment of evidence quality. Furthermore, to facilitate enhanced comparability of outcome associations, each correlation was recalibrated utilizing a random-effects model. We assessed the methodological quality of the included meta-analyses using a standard method (AMSTAR), and all the articles were of moderate or higher quality, with 55.2% being of high quality. However, a recent study in the cardiovascular field reported that the majority of systematic reviews were of "Critically low" (53%) or "Low" (18.7%) quality [81], which was contrast starkly with our findings. The reason for this inconsistent is that we use different tools for assessing the methodological quality of the included meta-analyses, and the quality assessments in the our study were relatively lenient (AMSTAR). To further improve the reliability of the results, we performed sensitivity analyses on meta-analyses that were excluded due to overlap and found that the certainty of most evidence remained unchanged. Moreover, our study included a large sample size with a comprehensive range of outcomes, and we presented them separately for different subgroups of the same outcome.

Potential limitations should be considered in our study. Firstly, the UR consisted entirely of meta-analyses of observational studies, which are subject to inherent limitations such as selection and confounding biases in the original articles. This may also be the reason for the low certainty of evidence. However, limited randomized controlled trials have examined the association between the TyG index and health outcomes. For example, one randomized controlled trial examined whether there were sex differences in the relationship between IR (evaluated by TyG index) and MACEs in hypertensive patients without diabetes [82]. The findings indicated an association between the TyG index and MACEs among hypertensive patients, with no observed differences in this association with respect to gender [82]. Nonetheless, a short follow-up time may limit the application of the results of this study. Hence, observational studies become more pertinent to our subject matter due to their advantages, such as larger sample sizes, a sufficient quantity of studies, and extended follow-up durations [83]. Secondly, the UR relied on previously published systematic reviews with meta-analyses. Although it is possible that some individual studies were missed, the use of a comprehensive search strategy and MeSH terms likely mitigated this risk. Thirdly, systematic reviews with only qualitative analyses and meta-analyses without study-specific data were excluded. Consequently, there is a potential for misestimation of these findings. Nevertheless, we summarized findings from these studies to ensure that we considered all relevant research. The majority (77.8%) of qualitative studies showed that increased TyG index raised the risk of atherosclerosis, MACE, and cerebrovascular disease, which was in line with our findings. (Additional file 1: Table S8). Moreover, several meta-analyses in our UR included fewer than 10 original studies. This may reduce the statistical power of Egger’s regression test [84] and make it difficult to assess the risk of publication bias.

## Conclusion

Although the TyG index is associated with many health outcomes, the high certainty evidence has been only observed for six associations, in which the TyG index is positively associated with the risk of CIN in non-diabetic patients with non-ST-segment elevation MI after PCI, the risk of stroke in patients with diabetes or ACS patients, the prognosis of CAD (non-fatal MI after PCI) and the severity of CAD (coronary artery stenosis and multivessel CAD). Therefore, there is a critical need for high quality meta-analyses of the association of TyG index with a wider range of health outcomes in the future.

### Supplementary Information

**Additional file 1.** **Table S1.** PRISMA checklist of items to include when reporting a systematic review or meta-analysis. **Table S2:** PRIOR Checklist. **Table S3.** Search strategy. **Table S4.** The list of the excluded records during the process of full-text review. **Table S5.** Description of 95 meta-analyses investigating the associations between Triglyceride-glucose index and health outcomes. **Table S6.** GRADE classification of quality of evidence. **Table S7.** Sensitivity analysis results of excluded meta-analyses due to overlap. **Table S8.** The summary results of meta-analyses excluded due to lack of data for quantitative synthesis.

## Data Availability

The datasets used and/or analysed during the current study are available from the corresponding author on reasonable request.

## Abbreviations

ACS: Acute coronary syndrome
AMSTAR: A Measurement Tool to Assess Systematic Reviews
CAD: Coronary artery disease
CI: Confidence intervals
CI-AKI: Contrast-induced acute kidney injury
CIN: Contrast-induced nephropathy
GRADE: Grading of Recommendations, Assessment, Development and Evaluation
HR: Hazard ratio
IR: Insulin resistance
MACE: Major adverse cardiovascular event
MI: Myocardial infarction
MOOSE: Meta-analyses of observational studies in epidemiology
OR: Odds ratio
PCAD: Premature cardiovascular artery disease
PCI: Percutaneous coronary intervention
PECOS: Population, Exposure, Comparison, Outcome, Study design
PRIOR: Reporting guideline for overviews of reviews of healthcare interventions
PRISMA: Preferred reporting items for systematic reviews and meta-analyses
RR: Relative risk
TyG: Triglyceride glucose
T2DM: Type 2 diabetes mellitus
UR: Umbrella review
